# A comprehensive analysis of the androgen receptor gene and risk of breast cancer: results from the National Cancer Institute Breast and Prostate Cancer Cohort Consortium (BPC3)

**DOI:** 10.1186/bcr1602

**Published:** 2006-09-20

**Authors:** David G Cox, Hélène Blanché, Celeste L Pearce, Eugenia E Calle, Graham A Colditz, Malcolm C Pike, Demetrius Albanes, Naomi E Allen, Pilar Amiano, Goran Berglund, Heiner Boeing, Julie Buring, Noel Burtt, Federico Canzian, Stephen Chanock, Françoise Clavel-Chapelon, Heather Spencer Feigelson, Matthew Freedman, Christopher A Haiman, Susan E Hankinson, Brian E Henderson, Robert Hoover, David J Hunter, Rudolf Kaaks, Laurence Kolonel, Peter Kraft, Loic LeMarchand, Eiliv Lund, Domenico Palli, Petra HM Peeters, Elio Riboli, Daniel O Stram, Michael Thun, Anne Tjonneland, Dimitrios Trichopoulos, Meredith Yeager, the Breast and Prostate Cancer Cohort Consortium

**Affiliations:** 1 Program in Molecular and Genetic Epidemiology, Epidemiology Department, Harvard School of Public Health, 677 Huntington Avenue, Boston, MA 02115, USA; 2 Channing Laboratory, Harvard Medical School, 181 Longwood Ave., Boston, MA, USA; 3 CEPH, Fondation Jean Dausset, 27 rue Juliette Dodu, 75010 Paris, France; 4 Keck School of Medicine, University of Southern California, East Lake Ave. Los Angeles, CA, 90089 USA; 5 Epidemiology and Surveillance Research American Cancer Society, 1599 Clifton Rd. NE, Atlanta, GA, 30329 USA; 6 Division of Cancer Epidemiology and Genetics, National Cancer Institute, Executive Blvd Rockville, MD, 20852 USA; 7 Cancer Research UK Epidemiology Unit, University of Oxford, Richard Doll Building, Old Road Campus Oxford, UK OX3 7LF; 8 Molecular and Nutritional Epidemiology Unit, Scientific Institute of Tuscany, 50131 Florence, Italy; 9 Department of Medicine, Lund University, 221 00 Lund, Sweden; 10 Department of Epidemiology, German Institute of Human Nutrition, Potsdam-Rehbruecke, Arthur-Scheunert-Allee 114-116, 14558 Nuthetal, Germany; 11 Division of Preventive Medicine, Brigham & Women's Hospital, Department of Medicine, Harvard Medical School, 900 Commonwealth Ave., Boston, MA 02215, USA; 12 Broad Institute at Harvard and the Massachusetts Institute of Technology, 7 Cambridge Center, Cambridge, MA 02142, USA; 13 Genomic Epidemiology Group, Division of Molecular Genetic Epidemiology, German Cancer Research Center, 69121 Heidelberg, Germany; 14 Core Genotyping Facility, National Cancer Institute, 8717 Grovemont Circle, Gaithersburg, MD 20892, USA; 15 INSERM, Institut Gustave Roussy, 39 rue Camille Desmoulins, 94805 Villejuif, France; 16 Dana-Farber Cancer Institute, Department of Medical Oncology, 44 Binney St., Boston, MA 02115, USA; 17 Department of Epidemiology, Harvard School of Public Health, 677 Huntington Ave,. Boston, MA 02115, USA; 18 Nutrition and Hormones Group, International Agency for Research on Cancer,150 Cours Albert Thomas, 69008 Lyon, France; 19 Epidemiology Program, Cancer Research Center, University of Hawaii, 1236 Lauhala St., Honolulu, HI 96813, USA; 20 Institute of Community Medicine, University of Tromsø, 9037 Tromsø, Norway; 21 Molecular and Nutritional Epidemiology Unit, Scientific Institute of Tuscany, 50131 Florence, Italy; 22 Julius Center for Health Sciences and Primary Care, University Medical Center Utrecht, 3508 Utrecht, The Netherlands; 23 Faculty of Medicine, Division of Epidemiology, Public Health and Primary Care, Imperial College, W2 1PG London, UK; 24 Institute of Cancer Epidemiology, Danish Cancer Society, Strandboulevarden 49, DK-2100 Copenhagen, Denmark; 25 Department of Hygiene and Epidemiology, School of Medicine, University of Athens, 75 Mikras Asias Str., 11527 Goudi, Athens, Greece

## Abstract

**Introduction:**

Androgens have been hypothesised to influence risk of breast cancer through several possible mechanisms, including their conversion to estradiol or their binding to the oestrogen receptor and/or androgen receptor (AR) in the breast. Here, we report on the results of a large and comprehensive study of the association between genetic variation in the *AR *gene and risk of breast cancer in the National Cancer Institute Breast and Prostate Cancer Cohort Consortium (BPC3).

**Methods:**

The underlying genetic variation was determined by first sequencing the coding regions of the *AR *gene in a panel of 95 advanced breast cancer cases. Second, a dense set of markers from the public database was genotyped in a panel of 349 healthy women. The linkage disequilibrium relationships (blocks) across the gene were then identified, and haplotype-tagging single nucleotide polymorphisms (htSNPs) were selected to capture the common genetic variation across the locus. The htSNPs were then genotyped in the nested breast cancer cases and controls from the Cancer Prevention Study II, European Prospective Investigation into Cancer and Nutrition, Multiethnic Cohort, Nurses' Health Study, and Women's Health Study cohorts (5,603 breast cancer cases and 7,480 controls).

**Results:**

We found no association between any genetic variation (SNP, haplotype, or the exon 1 CAG repeat) in the *AR *gene and risk of breast cancer, nor were any statistical interactions with known breast cancer risk factors observed.

**Conclusion:**

Among postmenopausal Caucasian women, common variants of the *AR *gene are not associated with risk of breast cancer.

## Introduction

The effects of testosterone activity in the breast are still unknown, showing both proliferative and anti-proliferative effects *in vitro *[1-3]. Levels of testosterone, which is produced in the ovaries, adrenal gland, and peripherally in adipose tissue, either change little or decline slightly after menopause [4-9]. In both pre- and postmenopausal women, circulating testosterone levels are associated with increased risk of breast cancer [10-17].

The androgen receptor (AR) protein exists as two isoforms, both arising from the same DNA sequence on the X chromosome (Xq11-q12). The shorter form of the AR protein lacks the N-terminal region, which is coded by exon 1. Within exon 1 is a tri-nucleotide CAG repeat. Although this polymorphism is associated with *AR *transactivation activity [18-21] and prostate cancer risk in some studies [22-28], no clear association has been shown with breast cancer risk [29-34]. The 3' UTR (untranslated region) of the *AR *contains sequence elements that bind to proteins involved in regulation of mRNA stability. This and other sequence-specific characteristics of *AR *mRNA, including putative function of the repeats in exon 1, have recently been reviewed [35]. The AR is expressed in the normal breast, as well as in primary and metastatic breast cancer tumours, and both the expression and protein levels are correlated with tumour invasiveness [36].

We hypothesised that inherited polymorphisms in genes related to sex steroid hormone synthesis, metabolism, and cell signaling could alter the function of these genes and the proteins they encode, therefore altering breast cancer risk; in this report, we present results for the AR. We used a haplotype-tagging approach, which aims to capture common variants in the *AR *gene. Here, we present these haplotypes and describe their association with breast cancer risk in a pooled analysis of nested case control studies from a large collaborative study, the Breast and Prostate Cancer Cohort Consortium (BPC3) [37], which includes 5,603 cases of breast cancer and 7,480 controls.

## Materials and methods

### Study population

The BPC3 has been described in detail elsewhere [37]. Briefly, the consortium includes five large well-established cohorts assembled in the U.S. and Europe which have both DNA samples and extensive questionnaire information (the American Cancer Society Cancer Prevention Study II [38], the European Prospective Investigation into Cancer and Nutrition [EPIC] cohort [39], the Harvard Nurses' Health Study [NHS] [40] and Women's Health Study [WHS] [41], and the Hawaii-Los Angeles Multiethnic Cohort [MEC] [42]). Most women in these cohorts, with the exception of the MEC, were Caucasians of U.S. and European descent. Breast cancer cases were identified in each cohort by self-report with subsequent confirmation of the diagnosis from medical records or tumour registries and/or from linkage with population-based tumour registries (method of confirmation varied by cohort). Controls were matched to cases by ethnicity and age and, in some cohorts, additional criteria (such as country of residence in EPIC).

### Genotyping

Coding regions of *AR *were sequenced in a panel of 95 advanced breast cancer cases from the MEC (19 of each ethnic group: African-American, Latino, Japanese, Native Hawaiian, and white). Thirty-two single nucleotide polymorphisms (SNPs) with minor allele frequency greater than 5% in any of the five ethnic groups or greater than 1% overall were selected from this resequencing as well as any SNP available in dbSNP to be used to select haplotype-tagging SNPs (htSNPs). These SNPs were genotyped in a reference panel of 349 healthy women from the MEC populations (including 70 whites) at the Broad Institute (Cambridge, MA, USA) using the Sequenom, Inc. (San Diego, CA, USA) and Illumina, Inc. (San Diego, CA, USA) platforms, and six htSNPs were selected to maximise R^2^_H _(a measure of correlation between SNPs genotyped and the haplotypes they describe) among Caucasians, using the method of Stram *et al*. [43]. Genotyping of the six htSNPs in the breast cancer cases and controls was performed in three laboratories (University of Southern California, Los Angeles, CA, USA; Harvard School of Public Health, Boston, MA, USA; and International Agency for Research on Cancer, Lyon, France) using a fluorescent 5' endonuclease assay and the ABI-PRISM 7900 for sequence detection (Taqman) (Applied Biosystems, Foster City, CA). Initial quality control checks of the SNP assays were performed at the manufacturer (Applied Biosystems); an additional 500 test reactions were run by the BPC3. Assay characteristics for the six htSNPs for *AR *are available on a public website [44]. Sequence validation for each SNP assay was performed and 100% concordance was observed [45]. To assess inter-laboratory variation, each genotyping centre ran assays on a designated set of 94 samples from the Coriell Biorepository (Camden, NJ, USA), showing completion and concordance rates of greater than 99% [45]. The internal quality of genotype data at each genotyping centre was assessed by typing 5%–10% blinded samples in duplicate or triplicate (depending on the study); the resulting concordance was greater than 99%. The genotyping success rate was 94% or greater for each of the six SNPs at each genotyping centre. No deviation from Hardy-Weinberg equilibrium was observed among the controls in each cohort (at the *p *< 0.01 level) for any given assay. An association among the exon 1 CAG repeat in *AR*, family history of breast cancer, and breast cancer risk was previously reported (1990–96 follow-up in the NHS, 617 cases and 960 controls [46]). The exon 1 CAG repeat was genotyped in an additional 376 cases and 540 controls from the NHS as well as 669 cases and 674 controls from the WHS, as previously described [46]. Given that there is no association between the CAG repeat and breast cancer risk, and the interaction between this polymorphism and family history was not observed in this larger combined sample set (1,662 cases and 2,174 controls), we decided not to expend the resources necessary to genotype the repeat in the remaining data sets.

### Statistical analysis

We used conditional multivariate logistic regression to estimate odds ratios (ORs) for disease in subjects with a linear (additive) scoring for zero, one, or two copies of the minor allele of each SNP. We also used conditional logistic regression with additive scoring and the most common haplotype as the referent to estimate haplotype-specific ORs, using an expectation-substitution approach to assign haplotypes based on the unphased genotype data and to account for uncertainty in assignment [47,48]. Haplotype frequencies and expected subject-specific haplotype indicators were calculated separately for each cohort (as well as by country within EPIC and race in the MEC). To test the global null hypothesis of no association between variation in *AR *haplotypes and htSNPs and risk of breast cancer (or subtypes defined by receptor status), we used a likelihood ratio test comparing a model with additive effects for each common haplotype (treating the most common haplotype as the referent) to the intercept-only model. We combined rare haplotypes (those with estimated individual frequencies less than 5% in all cohorts) into a single category that comprised less than 1.5% of the controls.

We considered both unadjusted conditional models and conditional models adjusting for known breast cancer risk factors. The covariates included to account for breast cancer risk factors were age at menarche (≤12 years, 13–14 years, 15+ years), menopausal status (pre-, peri-, and postmenopausal), parity (ever/never full-term pregnancy), body mass index (BMI) (in kg/m^2 ^as a continuous variable), and use of postmenopausal hormones (ever/never). Other common risk factors, including family history of breast cancer, personal history of benign breast disease, and age at menopause, were unavailable for large numbers of women and therefore were not included in the models. Because the results remained essentially unchanged regardless of the model used, we present results using the unadjusted conditional model. We also evaluated these covariates, restricting analyses of interaction to only those subjects with information available for variables such as family history, with categorical variables divided into quintiles. Interaction effects were evaluated using likelihood ratio testing, comparing models with the main effects of the genetic and risk variable to the model with these main effects and a multiplicative interaction term. Lastly, we tested whether the association between *AR *and breast cancer differed by menopausal status at diagnosis and tumour receptor (oestrogen receptor [ER] and progesterone receptor [PR]) status.

The exon 1 CAG repeat was analysed as previously reported [44]. Interaction *p *values between number of repeats and family history were calculated using likelihood ratio tests comparing the model with main effects for carrying at least one long repeat (cutoffs of ≥22, 23, 25, and 27 repeats) and family history with the model containing these main effects, and an additional interaction term, with homozyotes of the ≥22 allele with no family history as the reference.

## Results

**Figure 1:**
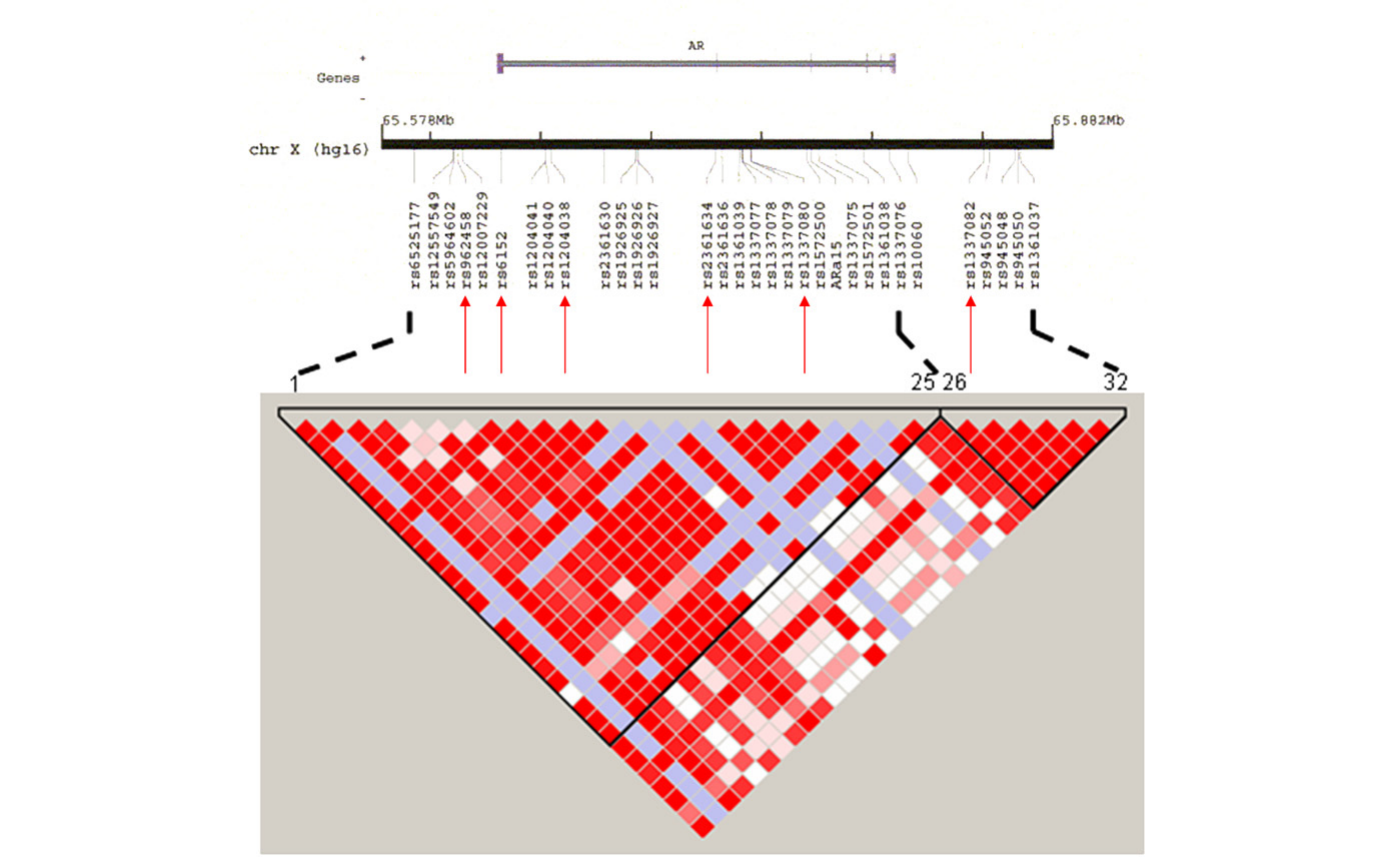
Linkage disequilibrium (LD) at androgen receptor (*AR*). Linkage disequilibrium plot generated by Haploview [50] showing correlation between all pairs of single nucleotide polymorphisms (SNPs) genotyped to select haplotype-tagging SNPs (ht-SNPs). The 32 SNPs genotyped among the 349 Multiethnic Cohort samples are shown in their physical position along the X chromosome (solid black horizontal line) and relative to the *AR *gene (depicted above the X chromosome plot). htSNPs selected are shown by arrows. Although two LD blocks are shown (black outline in LD plot), high correlation between the blocks allowed for the analysis of the htSNPs as one block.

Figure 1 shows the genomic structure of the region around *AR*. One very common (approximately 70%) haplotype exists, with six lower-prevalence haplotypes being defined each by the htSNPs. The minimum R^2^_H _for these six SNPs was 0.77 in the Japanese, white, and Latina samples from the SNP selection panel. However, these SNPs do not describe haplotype diversity among African-Americans (minimum R^2^_H _= 0.03).

A total of 5,603 cases and 7,480 controls were available for genotyping. Table 1 shows some of the baseline characteristics of these cases and controls. Genotyping success for each polymorphism was greater than 94%, and samples not yielding a genotype for a given SNP were removed from analyses for that SNP. Samples not yielding at least one genotype were removed from haplotype analyses, for a total of 5,584 cases and 7,459 controls. No associations with breast cancer or heterogeneity of risk estimates across the participating cohorts were observed for any individual SNP (Table 2) or haplotype tagged by these SNPs (Table 3). No differences in haplotype distribution were observed between ER^+ ^(*n *= 2,543) and ER^- ^(*n *= 590) cases (global *p *value = 0.61), PR^+ ^(*n *= 2,158) and PR^- ^(*n *= 860) cases (global *p *value = 0.51), or localised (*n *= 2,964) and metastatic (*n *= 1,646) cases (global *p *value = 0.43). No statistically significant interactions were observed between haplotypes and common breast cancer risk factors such as family history (yes/no), BMI (≤25, >25), age at first full-term pregnancy (nulliparous, ≤24, >24), or alcohol consumption (non-drinkers, ≤5 g/day, >5 g/day) (*p *interaction = 0.13, 0.16, 0.14, and 0.28, respectively). These results were not materially different after excluding African-American women from the MEC (344 cases and 426 controls).

Data from further follow-up of the NHS and the WHS did not support the previous findings of interaction between the *AR *CAG repeat and family history on breast cancer risk in the NHS with follow-up to 1996 [46]. No statistically significant interactions between longer *AR *CAG repeat length and positive family history were observed in either the further NHS follow-up or the WHS. The decrease in risk associated with shorter repeats among family history positive cases as previously reported [46] was not observed (Table 4).

## Discussion

One of the main aims of the BPC3 was to overcome limitations of prior studies by increasing sample size and, therefore, power of the study. By choosing genes involved in the synthesis, metabolism, and signaling of sex hormones, we aimed to maximise the possibility of finding alleles that predispose to breast cancer. Although the *AR *gene is a likely candidate gene, no association between polymorphisms in the *AR *gene and breast cancer risk was observed, despite the large sample size (5,603 cases and 7,480 controls) and systematic approach of this study.

In a previous study [46], shorter alleles of the CAG repeat polymorphism in exon 1 of the *AR *gene were associated with decreased risk of disease in women with a family history of breast cancer in the NHS. Adding samples from further follow-up cycles of the NHS, as well as samples from the WHS, we were unable to confirm this initial finding.

Mutations in genes such as *BRCA1 *(breast cancer 1, early onset) and *BRCA2*, although highly penetrant, are of low prevalence in the general population. Very few common polymorphisms have been shown to be associated with breast cancer risk. Using a candidate gene approach to select genes of possible interest in breast cancer etiology has also yielded very few breast cancer-susceptibility loci. One possible explanation for the lack of consistent association between common polymorphisms and breast cancer risk in individual studies is that the change in risk associated with common variants is too low to detect in individual studies and results that are reported may reflect publication bias.

The 95% confidence intervals in our study were narrow and exclude a substantial association between common variants in the *AR *gene with breast cancer risk. A concern that is more specific to the *AR *gene is that, due to the gene's location on the X chromosome, X chromosome inactivation could bias risk estimates associated with a causal allele toward the null. Such bias would be especially likely if the same X chromosome (either maternal or paternal) were inactivated in all breast tissue within each woman. However, X chromosome inactivation occurs very early in embryonic development and differs between lobes within the same breast [49]. Assuming a low-penetrance allele (as hypothesised here), women who are heterozygous for a putative risk allele on the X chromosome are still at approximately half the risk of developing breast cancer as women who are homozygous for the same allele, as approximately half (from random inactivation) of the breast cells would not express or be exposed to the risk allele, compared with all breast cells expressing the risk allele among homozygotes. This somewhat limits the possibility that X chromosome inactivation patterns could bias risk estimates toward the null. Optimally, tumour tissue from heterozygous women would be analysed to determine which allele is inactivated; however, this is not possible in the present study, because tumour specimens are not available.

Due to the low numbers of premenopausal women in our study, we cannot exclude the *AR *gene as a susceptibility locus for breast cancer occurring before menopause. Additionally, although the MEC does provide information from non-Caucasian individuals, there are not a sufficient number of samples, and htSNPs selected to describe genetic variation in Caucasians is not sufficient among African-Americans to definitively exclude polymorphisms in the *AR *gene as breast cancer-susceptibility alleles except in Caucasians. Among the latter, neither common variants nor the CAG repeat in exon 1 of the *AR *gene is associated with risk of postmenopausal breast cancer.

## Conclusion

Common polymorphisms in the *AR *gene are not associated with breast cancer risk among postmenopausal Caucasian women.

## Abbreviations

AR = androgen receptor; BMI = body mass index; BPC3 = Breast and Prostate Cancer Cohort Consortium; EPIC = European Prospective Investigation into Cancer and Nutrition; ER = oestrogen receptor; htSNP = haplotype-tagging single nucleotide polymorphism; MEC = Multiethnic Cohort; NHS = Nurses' Health Study; OR = odds ratio; PR = progesterone receptor; SNP = single nucleotide polymorphism; WHS = Women's Health Study.

## Competing interests

The authors declare that they have no competing interests.

## Authors' contributions

DGC, H Blanché, CLP, EEC, GAC, and MCP made up the writing committee for this work and were responsible for data analyses, manuscript preparation, and editing. NB and MF performed the htSNP selection and contributed substantially to manuscript editing. SC, FC, CAH, PK, DOS, and MY provided expertise in genotyping and results analyses, as well as manuscript editing. DA, NEA, PA, GB, H Boeing, JB, FC-C, HSF, SEH, BEH, RH, DJH, RK, LK, LL, EL, DP, PP, ER, MT, AT, and DT contributed substantially to sample collection and manuscript editing. All authors read and approved the final manuscript.

## Figures and Tables

**Table 1:** Baseline characteristics of BPC3 participants by cohort

	ACS		EPIC		MEC		NHS		WHS		All	
	Cases	Controls	Cases	Controls	Cases	Controls	Cases	Controls	Cases	Controls	Cases	Controls

Number	503	505	1,719	2,844	1,617	1,962	1,059	1,464	705	705	5,603	7,480
												
Mean age in years at blood donation (standard deviation)	68.9 (5.9)	68.9 (5.8)	55.4 (7.9)	55.1 (8.3)	66.4 (8.6)	63.6 (8.7)	57.6 (7.0)	58.2 (6.9)	56.0 (7.3)	56.0 (7.3)	60.2 (9.4)	59.0 (9.0)
												
Mean body mass index (standard deviation)	25.2 (4.6)	25.5 (4.6)	25.9 (4.4)	26.1 (4.6)	26.7 (5.6)	27.0 (6.0)	25.4 (4.7)	25.6 (4.7)	25.5 (4.4)	26.0 (5.1)	25.9 (4.9)	26.2 (5.1)
												
Age in years at menarche												
≤12	46.9	45.3	35.5	36.2	53.9	49.7	42.4	48.7	56.7	51.6	47.8	44.3
13–14	43.9	46.7	48.5	45.5	35.5	38.6	39.5	42.9	37.0	42.7	41.1	43.0
15+	9.3	8.0	16.0	18.4	10.5	11.7	8.1	8.4	6.2	5.7	11.0	12.7
												
Menopausal status												
Premenopausal	0	0	24.3	27.7	11.0	16.5	19.4	16.9	21.7	21.2	17.0	20.2
Postmenopausal	100	100	67.1	63.6	86.5	81.8	71.3	74.8	63.5	60.2	76.0	72.7
Perimenopausal/unknown	0	0	8.6	8.7	2.5	1.7	9.4	8.3	14.8	18.6	7.0	7.1
												
Age in years at menopause*												
<40	10.2	9.9	4.5	5.0	16.3	18.9	9.9	11.1	7.1	9.0	10.6	11.4
40–44	10.2	12.4	9.5	10.3	14.9	16.9	12.2	11.3	10.0	14.5	12.0	13.1
45–49	20.4	28.2	28.9	31.8	26.6	27.9	27.6	28.1	31.1	31.6	27.0	29.4
50–54	46.6	39.7	47.1	44.0	32.8	29.0	44.5	43.7	43.9	36.8	41.3	38.4
55+	12.6	9.9	10.0	8.9	9.4	7.3	5.8	5.8	7.8	8.0	9.1	7.8
												
Parous women	91.0	91.4	86.7	86.8	85.5	88.8	92.3	93.3	84.8	86.2	87.5	88.9
												
Family history	20.7	15.5	-	-	17.1	11.1	19.2	13.9	20.1	16.3	18.6	13.3
												
Ever HRT use*	67.0	58.7	42.3	34.1	63.9	58.9	77.2	72.0	68.0	60.3	61.4	53.5
Carcinoma *in situ*	21.5	-	6.3	-	1.0	-	0	-	0	-	4.1	-
ER-positive	52.7	-	-	-	62.0	-	68.0	-	80.4	-	65.8	-
PR-positive	44.5	-	-	-	51.1	-	57.5	-	71.9	-	55.8	-
												
Ethnicity												
White	97.8	98.6	100	100	24.8	22.4	93.6	93.6	95.6	95.6	76.3	77.9
Hispanic	0.8	0.2	0	0	20.8	19.7	0.1	0.3	0.6	0.6	6.2	5.3
African-American	0.8	0.8	0	0	21.3	21.7	0.8	0.6	0.7	0.7	6.5	5.9
Asian	0.4	0.4	0	0	26.3	21.4	0.1	0.4	1.0	1.0	7.8	5.8
Hawaiian	0	0	0	0	6.7	14.8	0	0	0	0	2.0	3.9
Other	0.2	0	0	0	0	0	5.5	5.1	2.1	2.1	1.3	1.2

ACS, American Cancer Society; BPC3, Breast and Prostate Cancer Cohort Consortium; EPIC, European Prospective Investigation into Cancer and Nutrition; ER, oestrogen receptor; HRT, hormone replacement therapy; MEC, Multiethnic Cohort; NHS, Nurses' Health Study; PR, progesterone receptor; WHS, Women's Health Study.

* Among post-menopausal women only

**Table 2:** Association between breast cancer and individual htSNPs in the *AR *across the BPC3 cohorts

SNP	Genotype	Cases (percentage)^a^	Controls (percentage)^a^	OR (95% CI)^b^	*P *trend^c^	*P *heterogeneity^d^
AR_001	AA	4,480 (82)	6,034 (83)	1.00 (Ref.)	.	.
rs962458	AG	826 (15)	1,054 (14)	1.04 (0.95 to 1.15)	.	.
	GG	146 (3)	187 (3)	1.02 (0.83 to 1.26)	0.50	0.96
AR_002	GG	3,833 (71)	5,073 (70)	1.00 (Ref.)	.	.
rs6152	GA	1,324 (25)	1,815 (25)	0.98 (0.90 to 1.06)	.	.
	AA	245 (5)	314 (4)	1.02 (0.87 to 1.20)	0.89	0.84
AR_003	GG	3,773 (70)	4,984 (69)	1.00 (Ref.)	.	.
rs1204038	GA	1,279 (24)	1,762 (24)	0.97 (0.90 to 1.06)	.	.
	AA	365 (7)	452 (6)	1.05 (0.89 to 1.23)	0.96	0.86
AR_004	AA	4,940 (90)	6,540 (90)	1.00 (Ref.)	.	.
rs2361634	AG	524 (9)	727 (10)	0.95 (0.85 to 1.07)	.	.
	GG	25 (<1)	24 (<1)	1.30 (0.76 to 2.21)	0.70	0.56
AR_005	AA	4,601 (84)	6,188 (85)	1.00 (Ref.)	.	.
rs1337080	AG	802 (15)	1,012 (14)	1.07 (0.97 to 1.18)	.	.
	GG	65 (1)	81 (1)	1.06 (0.80 to 1.41)	0.20	0.88
AR_006	AA	3,442 (63)	4,596 (63)	1.00 (Ref.)	.	.
rs1337082	AG	1,601 (29)	2,154 (29)	1.01 (0.94 to 1.09)	.	.
	GG	416 (8)	556 (8)	0.99 (0.85 to 1.14)	0.97	0.29

^a^Numbers of cases and controls genotyped for each SNP vary due to assay failure; ^b^unadjusted logistic regression, conditional on matching factors and cohort as described in text; ^c^test for trend for log-additive or codominant model; ^d^test for heterogeneity in ORs across cohorts. AR, androgen receptor; BPC3, Breast and Prostate Cancer Cohort Consortium; CI, confidence interval; htSNP, haplotype-tagging single nucleotide polymorphism; OR, odds ratio; Ref., Reference category; SNP, single nucleotide polymorphism.

**Table 3:** Association between AR haplotypes and breast cancer risk in the NCI BPC3

Haplotype	Percentage in cases (*n *= 5,584)	Percentage in controls (*n *= 7,459)	OR^a ^(95% CI)
AGGAAA	0.72	0.71	1.00 (Ref.)
GAAAGG	0.08	0.08	1.01 (0.93 to 1.11)
AAAAAG	0.06	0.07	0.94 (0.85 to 1.03)
AGGGAA	0.05	0.05	0.97 (0.87 to 1.08)
AGGAAG	0.05	0.05	1.02 (0.91 to 1.15)
GAAAAG	0.02	0.02	0.99 (0.83 to 1.18)
AGAAAG	0.02	0.01	0.98 (0.82 to 1.18)
<5%	0.02	0.01	1.18 (0.98 to 1.43)

^a^Unadjusted logistic regression, conditional on matching factors and cohort as described in text. AR, androgen receptor; CI, confidence interval; NCI BPC3, National Cancer Institute Breast and Prostate Cancer Cohort Consortium; OR, odds ratio; Ref., Reference category.

**Table 4:** AR exon 1 CAG Repeat length and family history in the NHS and the WHS

Family history	Genotype^a ^[CAG]_n_	1990–1996 OR (95% CI)^b^	1998–2000 OR (95% CI)^b^	WHS OR (95% CI)^b^	NHS and WHS Combined OR (95% CI)^b^
no	<22	1.0 (Ref.)	1.0 (Ref.)	1.0 (Ref.)	1.0 (Ref.)
no	≥22	0.9 (0.8 to 1.2)	1.0 (0.7 to 1.4)	0.7 (0.5 to 1.0)	1.0 (0.9 to 1.2)
yes	<22	1.0 (0.6 to 1.7)	2.0 (0.9 to 4.2)	1.6 (0.8 to 3.0)	1.1 (0.9 to 1.5)
yes	≥22	1.7 (1.2 to 2.4)	1.6 (0.9 to 2.6)	1.1 (0.7 to 1.8)	1.3 (1.1 to 1.6)

no	<22	1.0 (Ref.)	1.0 (Ref.)	1.0 (Ref.)	1.0 (Ref.)
no	≥23	0.9 (0.7 to1.2)	0.9 (0.7 to 1.3)	0.7 (0.5 to 1.0)	1.0 (0.9 to 1.2)
yes	<22	1.0 (0.8 to 1.4)	1.9 (0.9 to 4.1)	1.6 (0.8 to 3.0)	1.1 (0.9 to 1.5)
yes	≥23	1.8 (1.2 to 2.5)	1.5 (0.9 to 2.6)	1.0 (0.6 to 1.5)	1.4 (1.1 to 1.8)

no	<22	1.00 (Ref.)	1.0 (Ref.)	1.0 (Ref.)	1.0 (Ref.)
no	≥25	1.0 (0.8 to 1.4)	0.9 (0.6 to 1.3)	0.7 (0.5 to 1.1)	1.0 (0.9 to 1.2)
yes	<22	1.1 (0.6 to 1.8)	1.8 (0.9 to 4.0)	1.6 (0.8 to 3.0)	1.1 (0.9 to 1.5)
yes	≥25	2.0 (1.3 to 3.1)	1.5 (0.7 to 3.0)	0.7 (0.4 to 1.3)	1.5 (1.1 to 2.0)

no	<22	1.0 (Ref.)	1.0 (Ref.)	1.0 (Ref.)	1.0 (Ref.)
no	≥27	1.1 (0.7 to 1.7)	1.4 (0.8 to 2.5)	0.8 (0.5 to 1.3)	1.1 (0.9 to 1.4)
yes	<22	1.1 (0.6 to 1.9)	2.0 (0.9 to 4.6)	1.6 (0.8 to 3.0)	1.1 (0.9 to 1.6)
yes	≥27	1.6 (0.8 to 3.1)	2.1 (0.7 to 6.5)	0.7 (0.3 to 1.8)	1.4 (0.9 to 2.1)

^a^AR genotype defined as having one or more long alleles ≥22, 23, 25, or 27 versus both alleles <22; ^b^unconditional, unadjusted logistic regression. AR, androgen receptor; CI, confidence interval; NHS, Nurses' Health Study; OR, odds ratio; Ref., Reference category; WHS, Women's Health Study.
